# Knowledge of mental health symptoms and help seeking attitude in a population-based sample in Hong Kong

**DOI:** 10.1186/s13033-021-00462-2

**Published:** 2021-04-28

**Authors:** Ada Wai Tung Fung, Linda Chiu Wa Lam, Sandra Sau Man Chan, Sing Lee

**Affiliations:** 1 Department of Applied Social Sciences, The Hong Kong Polytechnic University, EF710, 7/F, Core E, Hung Hom, Hong Kong SAR, China; 2 Department of Psychiatry, The Chinese University of Hong Kong, G/F, Multicenter, Tai Po Hospital, Tai Po N.T, Hong Kong SAR, China

**Keywords:** Literacy, Mental health, General public, Barriers, Prevention, Psychiatry, Mood symptoms, Onset, Severity, Recognition

## Abstract

**Background:**

Mental health symptoms can be subtle, resulting in delaying treatment. A prompt identification of mental signs and symptoms is important for preventing mental disorders in the public. This study examined whether local public have adequate knowledge to identify mental health symptoms and the need to get timely professional help.

**Methods:**

The population-based telephone surveys were conducted in 2015 and 2018. It involved a random sample of 4033 respondents aged 12–75 years. Mental health knowledge and help seeking attitude were assessed using six vignettes depicting subtle and obvious symptoms of anxiety disorders, mixed anxiety and depressive disorders, and dementia. Logistic regression models were performed to examine association between mental health knowledge and help-seeking attitude.

**Results:**

Individuals with poor knowledge in subtle symptoms were more likely to be males (t =  − 5.0, p < .001), younger (F = 15.0, p < .001), have tertiary education (F = 15.0, p < .001), and employed (t =  − 2.1, p = .037). The knowledge scores of subtle and obvious symptoms were 1.5 and 2.3 respectively. Binary logistic regression found that poor knowledge of subtle symptoms was associated with reluctance to professional help seeking.

**Conclusions:**

Poorly identified subtle mental health symptoms is a major barrier to early professional help in highly educated working males. Future research should explore specific interventions to increase knowledge and professional help seeking in this group.

## Background

The Hong Kong Mental Morbidity Survey 2010–2013 reported that 13.3% of the population aged between 16 and 75 struggled with some types of common mental disorders, which is comparable to the global prevalence of 13% [1, 2]. A recent population-based survey further revealed that the proportion of people experiencing stress and anxiety symptoms has increased by 42.3%, and depressive symptoms and unhappiness have doubled compared to 2016 [3]. It is certain that subtle mental health symptoms are more common now than before, but they are not getting easier to be identified. Longitudinal evidence demonstrated that delaying treatment of up to 23 years could be resulted for any mood disorders [4]. Therefore, a prompt and accurate identification of mental signs and symptoms could be beneficial to the mental health in the public.

However, mental health literacy studies still found inaccurate labelling of mental disorders and low willingness to use mental health service in some developed countries [5–8]. Cross-cultural studies further revealed that identification of mental disorders in Asian populations is relatively low [9–11]. It is mainly due to inability to use psychiatric terms for subtle mental health symptoms and lack of knowledge in symptomatology [9]. Interestingly, a recent study comparing three cultural groups found that Hong Kong Chinese adults were comparable to British in terms of familiarity with mental illness terms compared to Malaysians, but the endorsement of the same population to professional help is still low. This reflects fair awareness but lack of knowledge in mental health disorders and associated burdens in local population [10].

There have been many studies on public awareness of mental health problems, but investigation on knowledge in symptomatology remained rudimentary and mostly focused on schizophrenia and depression in adolescences or young adults [12–16]. It is both timely and relevant to assess if the public have adequate knowledge to identify other common mental health disorders and the need to get professional help. We specifically looked at the level of knowledge in obvious and subtle symptoms of three common mental disorders, namely anxiety disorders, mixed anxiety and depressive disorder (MADD), and dementia, in a local population-based sample. We postulated that poor knowledge of subtle symptoms in these selected mental health disorders might be a major barrier to early professional help. We aimed to examine the extent of knowledge of mental health symptoms and to evaluate its association with help seeking attitude. Our study might provide a more comprehensive understanding of public mental health knowledge and advice effective delivery of public health education in the prevention of mental disorders under the current mental health crisis.

## Methods

### Study design and participants

A telephone survey was conducted with Computer Assisted Telephone Interview (CATI) at the Centre for Communication and Public Opinion Survey (CCPOS) of the Chinese University of Hong Kong (CUHK) in 2015 and 2018 respectively. A random sample of telephone numbers was drawn from a sampling frame generated from 2005, 2007 and 2009 Hong Kong residential number directory. The target respondents were land-based non-institutional Hong Kong residents aged 12–75 years, who spoke Cantonese, Putonghua or English. A random sample of 4033 respondents were successfully interviewed by trained CCPOS interviewers using a trilingual (Cantonese, Chinese and English) questionnaire. Foreign domestic helpers and those who were institutionalized were excluded from the study, as their daily activity pattern might be different and not be generalizable with the other Hong Kong residents. Verbal consent for all respondents and parental consent for those aged below 18 years were obtained over phone before interview.

### Vignettes

Six vignettes (V1–V6) were constructed to depict anxiety disorders in adolescents, MADD in adults and dementia in older adults. For each mental health condition, two scenarios were presented at two levels of difficulty with symptom descriptions. One case depicted more obvious mental symptoms with more constant intense feelings affecting daily function, while the other case described subtle symptoms of the same mental disorder that is either contextual or intermittent. Respondents were asked to identify symptoms as mental illnesses and recommend treatment for all vignettes. Please refer to Appendix or vignette’s description.

### Measurement

#### Knowledge of mental health symptoms

Knowledge of mental health symptoms was evaluated using the six vignettes. After the presentation of each vignette, respondents were asked to identify the vignette as 1 = Yes, he/she is likely to suffer from mental health disorders or 0 = No, he/she is not likely to suffer from mental health disorder/Do not know. A summary knowledge score ranges from 0 to 6, with higher score indicating higher level of knowledge.

#### Help seeking attitude on common mental health disorders

Help seeking attitude was explored based on the six vignettes described above. For each vignette, respondents were asked to opt for the most preferred help seeking methods: (i) nothing needs to be done; (ii) talking to family and friends; or (iii) consult a doctor or seek help from a professional. A total score for each help seeking attitude was calculated by summing the number of each methods chosen in all six vignettes. Each help seeking methods would score from 0 to 6, with higher score indicating higher preference towards a particular help seeking method.

#### Potential confounders

Mental health status was adjusted, as it could modulate the association between knowledge and help seeking attitude. It was assessed by the Short Warwick-Edinburgh Mental Well-Being Scale (SWEMWBS) and the Kessler Psychological Distress Scale (K6). K6 is a six-item questionnaire that measures psychological distress in the past 1 month anchoring on items: (i) feeling nervous, (ii) feeling hopeless, (iii) feeling restless or fidgety, (iv) feeling so depressed that nothing could cheer you up, (v) feeling everything was an effort, and (vi) feeling worthless. Each item is scored on a five-point Likert scale ranging from: (i) none of the time, (ii) a little of the time, (iii) some of the time, (iv) most of the time, and (v) all of the time. Higher scores, on a range of 0–24, indicate higher level of psychological distress. The K6 scale has a cut-off score at 13 to indicate severe psychological distress, with an internal reliability coefficient (Cronbach’s alpha) of 0.84 [17]. SWEMWBS is a validated seven-item instrument. It assessed the mental well-being status based on the frequency of experiencing seven positive feelings over the past two weeks: (i) feeling optimistic about the future, (ii) feeling useful, (iii) feeling relaxed, (iv) dealing with problems well, (v) thinking clearly, (vi) feeling close to other people, and (vii) able to make up own mind about things [18]. It is rated on a five-point Likert scale spanning from (i) none of the time, (ii) a little of the time, (iii) some of the time, (iv) most of the time, and (v) all of the time. It has a total score ranging from 7 to 35, with an internal reliability coefficient (Cronbach’s alpha) of 0.89. Higher scores indicate better mental well-being [18].

Sociodemographic information on age, gender, educational attainment, marital status, employment status, experience of mental health problems, and knowing someone with mental health problems were also obtained.

#### Statistical analysis

Descriptive statistics summarized the sociodemographic and mental health profiles of respondents. The overall knowledge of mental symptoms was determined by counting the number of vignettes successfully identified as mental illnesses. Similarly, knowledge on different types of symptoms (subtle and obvious) was determined by counting the number of vignettes successfully identified at each level. The demographic characteristics of those who could and could not identify mental health symptoms were compared to explore factors that might have associated with level of knowledge. Logistic regression models were performed to assess the association between help-seeking attitude and correct identification of mental health symptoms with adjustment for potential confounders. All data analyses were performed using IBM SPSS 23.0 for Windows. A p-value of < 0.05 was considered statistically significant.

## Results

### Sample characteristics

A total of 4033 respondents (42% males and 58% females) with a mean age of 46.1 (*SD* = 17.3) years. Table 1 displays the basic sociodemographic profile of the respondents. In terms of mental health status, the respondents had an average score of 5.6 (*SD* = 4.0) and 18.8 (*SD* = 4.4) on K6 and SWEMWBS respectively. 5.9% of the respondents experienced severe psychological distress (above K6 cut-off) during the past 2 weeks. There were 10.4% of the respondent with personal experience of mental health problem, and more than half (56.9%) of the respondents reported knowing Someone with mental health problems.

**Table 1:** Basic sociodemographic characteristic of the respondents

Sociodemographic variables	Overall (N = 4033)	
	Mean	SD
Age	46.1	17.3
12–17 (%)	3.7	–
18–44 (%)	38.5	–
45–64 (%)	40.4	–
65–75 (%)	17.4	–
Female (%)	58	–
Education attainment		
Primary level or below	10.3	–
Secondary level	41.9	–
Tertiary level	47.9	–
Marital status		
Single (%)	31.5	–
Married (%)	59.3	–
Divorced (%)	4.9	–
Widowed (%)	4.3	–
Employment status		
Working (%)	55.3	–
Not working (%)	27	–
Retired (%)	17.7	–
K6 score (Range 0–28)	5.6	4.0
≥ 13 (%)	5.9	–
SWEMWBS score (Range 0–35)	18.8	4.4
Personal experience of mental health problems (%)	10.4	–
Knowing someone with mental health problems (%)	56.9	–

*K6* Kessler psychological distress scale (K6), *SWEMWBS* Short Warwick-Edinburgh mental well-being scale

### Public knowledge of mental health disorders

Respondents had a mean score of 3.7 (*SD* = 1.5) out of 6 for overall knowledge of mental health symptoms. Only 11.1% of the respondents could identify symptoms in all six vignettes. The mean knowledge scores of subtle and obvious symptoms were 1.5 (*SD* = 0.9) and 2.3 (*SD* = 0.9) out of 3 respectively. Respondents were less likely to identify subtle symptoms than to identify obvious symptoms as mental disorders. Majority of them correctly identified 78.4% of anxiety disorders, 71.4% of MADD, and 76.7% of dementia as mental illnesses in vignettes with obvious symptoms. In contrast, a smaller proportion identified only 59.7% of anxiety disorders, 36.1% of MADD, and 48.9% of dementia as mental illnesses in the vignettes with subtle symptoms.

### Socio-demographic correlates of poor mental health knowledge

Independent t-test and ANOVA analysis were performed to explore whether there are significant differences in knowledge score based on a variety of sociodemographic factors. Individuals with lower knowledge score are more likely to be males (*t* =  − 5.0, *p* < 0.001), have younger age (*F* = 15.0, *p* < 0.001), have higher education (*F* = 15.0, *p* < 0.001), and employed (*t* =  − 2.1, *p* = 0.037). With respect to age and education, post-hoc comparison further indicated that the mean knowledge score was lowest in younger adults (Age 18–44) and those with tertiary education. Table 2 showed comparisons of knowledge scores on common mental health problems by gender, age and other socio-demographic variables of the respondents.

**Table 2:** Comparisons of knowledge scores on common mental health problems by sociodemographic variables of the respondents

Variable	Knowledge score		T-test/ANOVA
	Mean	SD	p-value
Gender			
Male	3.6	1.5	< .001
Female	3.8	1.4	
Age			
12–17	3.4	1.2	
18–44	3.6	1.3	
45–64	3.8	1.5	< .001
65–75	3.9	1.6	
Education			
Primary or below	4.0	1.5	< .001
Secondary	3.8	1.5	
Tertiary or above	3.6	1.4	
Marital status			
Married	3.7	1.5	1.74
Not married	3.7	1.3	
Employment status			
Working	3.7	1.4	.037
Not working	3.8	1.4	

Working males with tertiary education had a significantly lower overall knowledge score compared to the general public (3.5 ± 1.5 vs. 3.8 ± 1.4, *t* = 3.9, *p* < 0.001). In addition, their knowledge score of subtle mental health symptoms was significantly lower than the general public (1.3 ± 1.0 vs. 1.5 ± 0.9, *t* = 1.3, *p* < 0.001). No difference was found in mean knowledge score of obvious symptoms between the two groups (2.2 ± 0.9 vs. 2.3 ± 0.9, *t* = 1.9, *p* = 0.06).

### Attitude regarding professional help seeking

The mean attitude score towards professional help seeking was 3.7 (*SD* = 1.6) out of 6. Less than a fifth (15.9%) had a full score of 6 on professional help seeking. With regards to the subtle and obvious symptoms in anxiety vignettes, 56% and 66% of the respondents recommended consulting a doctor or seeking help from other professionals respectively; 35.5% and 29.7% of the respondents recommended talking to family and friends; and 8.6% and 3.5% recommended doing nothing respectively. For MADD, 69.6% (subtle) and 59.7% (obvious) of the respondents recommended consulting a doctor or seeking help from other professionals, 22.1% (subtle) and 36.5% (obvious) of the respondents recommended talking to family and friends and 8.9% (subtle) and 4% (obvious) recommended doing nothing. For dementia, 49.8% (subtle) and 68.6% (obvious) of the respondents recommended consulting a doctor or seeking help from other professionals, 27.6% (subtle) and 28.2% (obvious) of the respondents recommended talking to family and friends and 22.7% (subtle) and 3.3% (obvious) recommended doing nothing.

### Association between mental health knowledge and help seeking attitude

Bivariate correlation showed that mental health knowledge was correlated with professional help seeking attitude score (*r* = 0.4, p < 0.001), meaning that those who were more knowledgeable were more likely to have a good attitude towards professional help seeking. Linear regression analysis was performed to confirm the association (*B* = 0.5, 95% *CI* 0.4–0.5, *p* < 0.001) after adjusting for age, gender, educational attainment and employment status. More specifically, binary regression examined professional help seeking attitude in individuals with poor mental health knowledge. They were less likely to recommend professional help in most of the mental health conditions (subtle symptoms of anxiety: *OR* = 0.7, 95% *CI* 0.6–0.8, *p* < 0.001; subtle symptoms of MADD: *OR* = 0.7, 95% *CI* 0.6–0.9, *p* = 0.0001; subtle symptoms of dementia: *OR* = 0.6, 95% *CI* 0.5–0.7, *p* < 0.001; obvious symptoms of anxiety: *OR* = 0.8, 95%, *CI* 0.6–0.9, *p* = 0.002; obvious symptoms of MADD: *OR* = 0.7, 95% *CI* 0.6–0.8), *p* < 0.001). No association was found in dementia with obvious symptoms (*OR* = 1.1, 95% *CI* 0.9–1.3, *p* = 0.29).

## Discussion

The present survey was to examine the extent of mental health knowledge in specific symptoms identification and to evaluate its association with help seeking attitude on anxiety disorders, MADD and dementia, in a local populations-based sample. Our study found that over 70% of the respondents could identify obvious mental health symptoms on the three common mental disorders, but a comparatively lower rate of around 60% identified subtle mental health symptoms as mental illnesses. In addition, only 11% of respondents had sufficient knowledge to identify all mental health problems across vignettes. The findings does not only highlights the low rate of mental health knowledge in our local public, but also reveals a poor proficiency in identifying subtle mental health symptoms compared to identifying obvious mental health symptoms. This may reflect the underlying cause of poor mental health knowledge in Asians by which many might not recognize subtle symptoms as relevant or simply accepted as normal [19, 20].

We further demonstrated that working males with tertiary education were associated with lower knowledge score of overall mental health symptoms as well as subtle mental health symptoms. Apart from underestimation and tolerance of these subtle mental health symptoms at workplace among highly educated males, it might also reflect the presence of other deep-rooted barriers relating to cultural belief and stigmas against mental illness [21, 22]. For one, the traditional Confucianism might still profoundly influence the societal expectation of masculinity and family solidarity in local populations. The idea that men should be the breadwinner of their family is particularly detrimental to men than their female peers experiencing the same difficult times such as economic recession and COVID-19, given competing wages due to increasing education and employment opportunities in females. Second, disclosure of mental health problems relating to overwhelming family responsibility or relationship breakdown might be viewed as a taboo or weakness to masculinity and professional image among highly educated working males.

As hypothesized, we found that poor knowledge of subtle mental health symptoms was consistently associated with higher reluctance to professional help seeking. Indeed, previous cross-cultural study has already reported the tendency of reluctance to seek professional help and a higher likelihood of endorsing social support and self-help in Hong Kong Chinese adults [10]. This preference on self-reliance might be related to long existing stigma towards mental health disorders and negative experience with mental health professionals [20]. Another explanation might be due to the accessibility and affordability of professional services. In Hong Kong, traditional use of herbal medicines is much cheaper and more readily accessible than specialist consultation. In addition, it is commonly endorsed as a natural healing remedy for physical as well as mood related illnesses across age groups locally. Taken together, each might partly contribute to the reluctance of professional help seeking in local community.

### Strengths and limitations

A major strength of the study was the assignment of scoring to assess knowledge of mental health symptoms. This enabled comparison within and between subtle and obvious symptoms presentations of different mental health disorders. However, our study has several limitations. The sample consists of Hong Kong residents primarily, and thus generalizability of findings to other countries is limited. Second, cross-sectional study design could not make any causal direction on the association between poor knowledge of identifying subtle mental health symptoms and help seeking preference. Currently, we only focused on individual knowledge level, but many explanatory cultural and structural factors on reluctance to professional help seeking were not assessed in our study. Furthermore, the study was completed 2 years ago. The widespread mental health deterioration resulting from long anti-pandemic fatigue might likely to increase awareness as well as knowledge of mental health symptoms as more lay discussion and daily media coverage on the outbreak.

## Conclusions

The survey result suggests significant implication to increase mental health knowledge on subtle symptoms particularly in highly educated working males. While public campaigns may help to enhance acknowledgment of a wide range of mental health symptoms, more specific mental health information and intervention should be targeted at this hidden group for more sustained and significant impact on attitude change and knowledge enhancement. Future public mental health education might also start with male workers at managerial level, as they might have a higher chance to neglect subtle mental health symptoms and less motivated to seek help from mental health professional delaying timely treatment. At the same time, employers and firms might consider organizing group events or mental health talks to enhance workplace mental health knowledge and to provide a supportive working environment for the wellbeing of their employees. Furthermore, it might be useful to place more mental health hotline promotions in sports and drinking facilities where the highly educated working males most likely to go.

## Data Availability

The dataset generated and/or analyzed during the current study are not publicly available due to privacy but are available from the corresponding author on reasonable request.

## Appendix

### Vignette’s descriptions (V1–V6)

V1 Subtle symptoms of anxiety disorders: A 14-year-old adolescent boy whose academic performance is very good. But every time when he speaks in front of his teacher and classmates, he gets blushed, hand shivering, heart pounding and the mind gone blank. He is also afraid of being teased by others.

V2 Obvious symptoms of anxiety disorders: A 16-year-old adolescent girl who studies very hard and prepares well before class. But every time before examination, she has insomnia, abdominal pain, diarrhea and sweating, and thus resulting in poor examination results.

V3 Subtle symptoms of MADD: A 34-year-old working man who recently sweat easily, has a stiff neck and shoulder pain, and does not sleep well.

V4 Obvious symptoms of MADD: A 42-year-old housewife who always worries that her son would have accidents if he goes out. Her mind is full of negative thoughts.

V5 Subtle symptoms of dementia: A 68-year-old retired person who keeps reminiscing his wife about his past in the past year. He can clearly remember events that had happened long time ago.

V6 Obvious symptoms of dementia: A 75-year-old elderly lady who has left a boiling pot on the stove at home to the point where it was almost on fire many times.
